# Allopurinol Reduces the Lethality Associated with Acute Renal Failure Induced by *Crotalus durissus terrificus* Snake Venom: Comparison with Probenecid

**DOI:** 10.1371/journal.pntd.0001312

**Published:** 2011-09-06

**Authors:** Rodrigo Frezzatti, Paulo Flavio Silveira

**Affiliations:** Laboratory of Pharmacology, Instituto Butantan, São Paulo, São Paulo, Brazil; University of Kelaniya, Sri Lanka

## Abstract

**Background:**

Acute renal failure is one of the most serious complications of envenoming resulting from *Crotalus durissus terrificus* bites. This study evaluated the relevance of hyperuricemia and oxidative stress and the effects of allopurinol and probenecid in renal dysfunction caused by direct nephrotoxicity of *C. d. terrificus* venom.

**Methodology/Principal Findings:**

Hematocrit, protein, renal function and redox status were assessed in mice. High ratio of oxidized/reduced glutathione and hyperuricemia induced by *C. d. terrificus* venom were ameliorated by both, allopurinol or probenecid, but only allopurinol significantly reduced the lethality caused by *C. d. terrificus* venom. The effectiveness of probenecid is compromised probably because it promoted hypercreatinemia and hypocreatinuria and worsed the urinary hypo-osmolality in envenomed mice. In turn, the highest effectiveness of allopurinol might be due to its ability to diminish the intracellular formation of uric acid.

**Conclusions/Significance:**

Data provide consistent evidences linking uric acid with the acute renal failure induced by *C. d. terrificus* venom, as well as that this envenoming in mice constitutes an attractive animal model suitable for studying the hyperuricemia and that the allopurinol deserves to be clinically evaluated as an approach complementary to anti-snake venom serotherapy.

## Author Summary

In Brazil, among registered snake bites, those by the genus *Crotalus* originate the highest mortality rate. The rattlesnake *Crotalus durissus terrificus* is the most frequently implicated in these accidents. The kidney is a particularly vulnerable organ to the venom of this rattlesnake. In fact, the most serious complication of *Crotalus* snake bite is the renal dysfunction, and among the fatal cases of *Crotalus* bites in Brazil 5% are patients treated with antivenom. The hyperuricemia has been observed in human accidents with snake venoms, but this parameter has not received any special attention as a relevant factor in the etiology of renal dysfunction caused by these venoms. This study examined the effects of treatments with low-cost and low-risk uricostatic (allopurinol) and uricosuric (probenecid) drugs on the envenomation by *C. d. terrificus*, showing that allopurinol and probenecid mitigated certain nephrotoxic effects, as well as the survival of envenomed mice was improved through the effects of allopurinol on reduction of oxidative stress and intracellular formation of uric acid. This new knowledge provides consistent evidences linking uric acid with the renal dysfunction induced by rattlesnake bites and that the allopurinol deserves to be clinically evaluated as an approach complementary to anti-snake venom serotherapy.

## Introduction

Envenoming resulting from snake bite is recognized nowadays as one of the major neglected public health issue within poor communities living in the rural areas of several countries throughout the world. Because of serious misreporting, the true worldwide burden of snake bite is not known, but according to conservative country estimates used to calculate the regional estimates, Brazil had a fourth estimated number of envenomings annually (30,000) [1]. In Brazil, bites by snakes of genus *Crotalus* were responsible for 7.7% of these, but accidents involving this genus originate the highest mortality (about 2%) among registered snake bites [2]. Among the species of this genus, *Crotalus durissus terrificus* is the most frequently implicated in these accidents [3]. Because it is well vascularized, the kidney is a particularly vulnerable organ to venom toxicity [4]. In fact, the most serious complication of *Crotalus* snake bite is acute renal failure (ARF) [5]. The prospective survey of 100 cases of *Crotalus* bites followed from hospitalization to death or discharge in São Paulo, Brazil, revealed a high prevalence of ARF (29%) in the first 72 hours after the bite with the case fatality ratio of 10% [5].

Two primary events of the direct nephrotoxic effect of the *C. d. terrificus* venom (*vCdt*) in mice are the oxidative stress in renal tissue and an incidence of 100% of hyperuricemia over an incidence of 60% of hypercreatinemia [6]–[7]. Although hyperuricemia has also been observed in human accidents with *C. d. terrificus* [8] and others species of snakes [9], this parameter has not received any special attention as a relevant factor in the etiology of ARF, mainly because according to the recommendation of AKIN (Acute Kidney Injury Network) the prominent criteria to identify ARF should be the detection of changes in absolute values of serum creatinine, plasma urea and urinary volume [5], [10]–[12]. However, it is well known that in many situations ARF is associated with a rise in plasma uric acid as a result of both increased generation and decreased excretion [13]. A marked hyperuricemia, currently defined as uricemia higher than the maximum value of normal range [14]–[15], occurs with intrarenal urate crystal deposition leading to ARF [16]–[17], but rats with uricemia near three times higher than the minimum value of normal range (mild hyperuricemia) [14]–[15], as that caused in mice by *vCdt* [6]–[7], also develop systemic hypertension, interstitial renal disease, afferent arteriolopathy, increased renin expression [18]–[19] and glomerular hypertrophy [20]. Experimentally, raising uric acid in rats can induce these dysfunctions via a crystal-independent mechanism [21]–[25]. It has also been reported that mild hyperuricemia in rats can induce oxidative stress in the kidney [15] and within the endothelial [21] and vascular smooth muscle cells [26], as well as in adipocytes [27]. However, paradoxically the uric acid has been considered as circulating antioxidant which potently reacts with superoxide anion, peroxynitrite, chelates iron-based radicals [28] and prevents the oxidative inactivation of extracellular superoxide dismutase [29]. High plasma levels [30] or the infusion of uric acid [31] increased plasma antioxidant activity in humans.

Regarding the controversy about the effects of uric acid on redox status, the involvement of uric acid and oxidative stress in ARF induced by *vCdt* is an attractive reason to investigate this envenomation. Moreover, clinical investigations have established that antivenoms are highly effective in the neutralisation of toxins responsible for systemic effects, but among the fatal cases of *Crotalus* bites in Brazil 5% are patients treated with antivenom [32]. Therefore, to know more about the role of uric acid in ARF and to highlight potential complementary agents for the treatment of envenoming by *Crotalus* snake bites, this study evaluated the effects of uricostatic (allopurinol) and uricosuric (probenecid) drugs on renal function (hematocrit, protein, osmolality, creatinine, uric acid and urea) and oxidative stress (oxidized [GSSG] over reduced [GSH] glutathione index and content of malondialdehyde [MDA]) in mice inoculated with *vCdt*.

## Methods

### Ethics statement

The conducts and procedures involving animal experiments were approved by the Butantan Institute Committee for Ethics in Animal Experiments (License number CEUAIB 717/2010) in compliance with the recommendations of the National Council for the Control of Animal Experimentation of Brazil (CONCEA).

### Preparation of venom solution

1.0 mg of lyophilized venom (provided by the Instituto Butantan) was suspended in 1.0 mL of sterile phosphate buffered saline (PBS) (Na_2_HPO_4_.7H_2_O, 19.3 g/L; NaH_2_PO_4_.H_2_O, 3.9 g/L; NaCl, 8.77 g/L; pH 7.4), under mild mixing, for 10 min, at 4°C and, then, centrifuged at 10,192× *g* for 20 min at 4°C. The pellet was discarded and the supernatant was aliquoted and stored at −20°C at a maximum time of one week and administered intraperitoneally (ip) at a dose of 1.024 µg venom/20 g body mass (80%LD50) in a maximum volume of 0.2 mL [6]–[7]. The same lot of venom was used throughout this study.

### Preparation of allopurinol

Allopurinol (4-Hydroxypyrazolo[3,4-d]pyrimidine) (Sigma, USA) was dissolved in 1 M NaOH at a concentration of 50 mg/mL and subsequently diluted (1∶5) in PBS just before administration by gavage *per oral* (po) at a dose of 2 mg/20 g body mass in a maximum volume of 0.2 mL.

### Preparation of probenecid

Probenecid (*p*-[Dipropylsulfamoyl]benzoic acid) (Sigma, USA) was dissolved in 1 M NaOH at a concentration of 600 mg/mL and subsequently diluted (1∶5) in PBS just before administration po at a dose of 24 mg/20 g body mass in a maximum volume of 0.2 mL.

### Animals, treatments and urine collection

Adult male Swiss mice, weighing 18–20 g, provided by the Animal Facility of the Instituto Butantan, were maintained in polyethylene cages (inside length×width×height = 56×35×19 cm) with food and water *ad libitum*, in a container with controlled temperature of 25°C, relative humidity of 65.3±0.9% and 12 h ∶12 h photoperiod light∶ dark (lights on at 6:00 am).

Animals were divided into eight groups, which received: (i) 0.2 mL PBS, ip (control ip); (ii) 0.2 mL PBS, po (control po); (iii) 0.2 mL PBS, ip and after 2 h, 0.2 mL PBS, po (control ip+po); (iv) 2 mg allopurinol in 0.2 mL PBS per 20 g body mass, po (NL); (v) 24 mg probenecid in 0.2 mL PBS per 20 g body mass, po (PB); (vi) 1.024 µg venom in 0.2 mL PBS per 20 g body mass, ip (80%LD50) (*vCdt*); (vii) 80%LD50 *vCdt*, ip and after 2 h, 2 mg allopurinol in 0.2 mL PBS per 20 g body mass, po (*vCdt*+NL); (viii) 80%LD50 *vCdt*, ip and after 2 h, 24 mg probenecid in 0.2 mL PBS per 20 g body mass, po (*vCdt*+PB). Immediately after treatments, each group was placed in appropriate metabolic cages for urine collection, which was performed 24 h after venom injection. Pooled urine was centrifuged at 2,564× *g*, for 5 min, at 4°C; the supernatant was stored at −80°C, for the appropriate procedures. Immediately after urine collection, animals were anesthetized for blood and kidneys collection.

### Lethality

It was monitored at intervals of one hour for 24 h, starting after administration of venom or vehicle or drugs alone.

### Obtaining kidneys and plasma, and measurement of hematocrit

The animals were anesthetized with xylazin (Calmiun, Agener União, Brazil) 0.1% and ketamine (Cetamin, Syntec, Brazil) 1% (ip, 0.1 mL/10 g body mass). Then, the blood was collected with heparinized Pasteur pipette after scission in right axillary plexus. The thoracic cavity was opened to perform cardiac perfusion with 50 mM PBS, over a period of 5 min at a flow rate of 8–10 mL/min. Immediately after perfusion, kidneys were removed, frozen in dry ice and stored for a maximum period of 10 days, at −80°C, until the use in the appropriate procedures. Measurement of hematocrit was made in duplicate of individual samples, in micro-hematocrit capillary tubes, centrifuged at 3,000 rpm for 5 min, at room temperature (centrifuge HT model H240). For plasma obtainment, blood was centrifuged individually at 5,232× *g* for 5 min, at 4°C.

### Total protein

Total protein was measured, photometrically (Bio-Tek Power Wave XR spectrophotometer), at 630 nm, in triplicates, in samples of plasma (diluted 500-fold) and pool of urine (diluted 75-fold), by the method of Bradford [33], using a Bio-Rad protein assay reagent (Hercules, USA). Protein contents were extrapolated by comparison with standard curves of bovine serum albumin (BSA) in the same diluent.

### Osmolality, creatinine, uric acid and urea

The measurements were performed as described by Marinho et al. [34]. Briefly, osmolality was determined in triplicates of 10 µL with a cryoscopic osmometer (Osmette II Fisher) and creatinine, uric acid, and urea were photometrically quantified in triplicate of individual plasma and pooled urine samples.

### Oxidative stress

Oxidative stress was evaluated on renal cortex and medulla from dissected kidneys stored at −80°C.

GSSG and GSH were fluorometrically measured as described by Yamasaki et al. [6]. For this purpose, cortex and medulla were homogenized in 0.1 M phosphate buffer, with 0.005 M EDTA, pH 8.0 (PBEDTA) plus 5.26% HPO_3_ (0.1 g tissue/1.5 mL PBEDTA plus 0.4 mL 25% HPO_3_), at 800 rpm for 3 min. These homogenates were ultracentrifuged at 100,000× *g* for 30 min. The pellet was discarded and the supernatant was immediately used. All steps were carried out at 4°C.

MDA was assayed based on the method described by Selmanoglu et al. [35]. The reaction solution was prepared with 90 µL of 8.1% SDS; 675 µL of 20% acetic acid solution (pH 3.5 adjusted with NaOH); and 675 µL of 0.8% aqueous solution of thiobarbituric acid. 90 µL of tissue homogenates (10% [0.1 g/mL] prepared in 1.15% KCl) was added to this solution and then the volume was made up to 1.8 mL with distilled water. This mixture was kept in a 98°C dry bath for about 1 h and subsequently centrifuged at 2,500× *g* for 10 min at 4°C. The supernatant was measured at 532 nm. The 1,1,3,3-tetraethoxypropane was used as a standard for MDA.

### Histology

The kidneys were fixed in 4% paraformaldehyde solution in 0.1 M NAOH and 0.1 M sodium tetraborate, processed in the routine histological processes, 10-µm-thick sagittal sections were prepared and stained with hematoxylin and eosin for light microscopy examination.

### Statistical analysis

Data are shown as mean ± standard error of the mean (SEM) and were analyzed using GraphPad Prism™ software packages. Regression analyses were performed to obtain standard curves of protein, 1,1,3,3-tetraethoxypropane, GSH and GSSG. One-way analysis of variance (ANOVA) followed, when differences were detected, by the Newman-Keuls test was performed to compare values among groups. Lethality data were analyzed by the two-sided Fisher's exact test. In all the calculations, a minimum critical level of p<0.05 was set.

## Results

### Standardization of controls

Considering that various checked controls (ip, po and ip+po) presented statistically similar values for mortality, creatinine, uric acid and urea, the group that received vehicle (PBS) by ip and po routes (group ip+po), both routes of administration of venom and drugs, was adopted as control.

### Lethality

Table 1 shows that the lethality of 80%LD50 of *vCdt* (43%) at 24 h was about the theoretically expected (40%) and did not differ statistically from that of the *vCdt* group treated with probenecid (25%). The lethality rate at 24 h was null in all control groups and in those treated with allopurinol or probenecid alone, as well as significantly reduced about 58% (from 43% to 18%) in *vCdt* group treated with allopurinol. Most deaths occurred between 14–16 h and fewer between 16–18 h in envenomed mice. In envenomed mice treated with probenecid most deaths occurred between 16–18 h while in envenomed mice treated with allopurinol occurred between 18–20 h.

**Table 1:** Assessment of lethality after treatments.

Treatments	Number of mice	Time course (h) of lethality rate (%)					
		0–14	14–16	16–18	18–20	20–23	23–24
**control ip**	5	0	0	0	0	0	0
**control po**	6	0	0	0	0	0	0
**control ip+po**	5	0	0	0	0	0	0
**NL po**	9	0	0	0	0	0	0
**PB po**	10	0	0	0	0	0	0
***vCdt*** ** ip**	28	0	29	14	0	0	43*
***vCdt*** ** ip+NL po**	40	0	0	3	15	0	18
***vCdt*** ** ip+PB po**	36	0	3	16	6	0	25*

Mice treated intraperitoneally (ip) and/or *per oral* (po) with vehicle (control), allopurinol (NL), probenecid (PB) and *Crotalus durissus terrificus* venom (*vCdt*) followed by NL (*vCdt*+NL) or PB (*vCdt*+PB) after 2 h.

*Lethality at 24 h only differed in *vCdt*+PB (p = 0.0432) and *vCdt* (p = 0.0016) compared with all other groups. Lethality at 24 h did not differ between *vCdt*+PB and *vCdt* (p = 0.1811) (Two-sided Fisher's exact test).

### Renal function parameters

**Figure 1:**
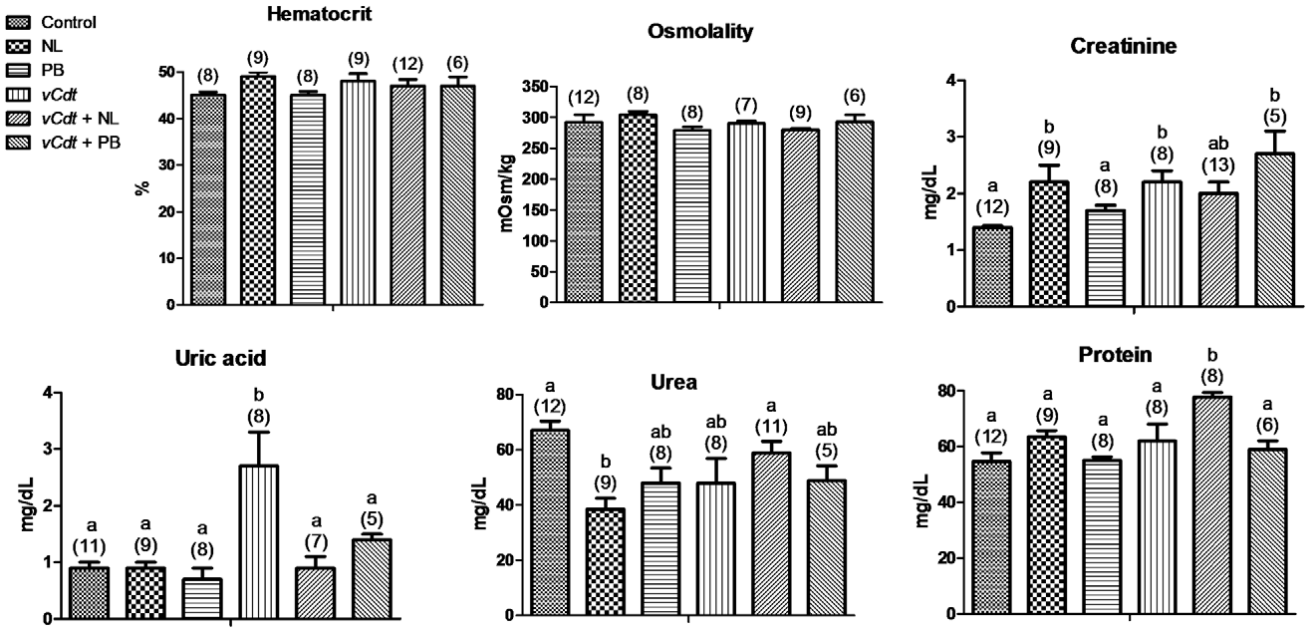
Effects of treatments on hematocrit and renal function parameters in the blood plasma. Mice treated with vehicle (control), allopurinol (NL), probenecid (PB) and *Crotalus durissus terrificus* venom (*vCdt*) followed by NL (*vCdt*+NL) or PB (*vCdt*+PB) after 2 h. Values are means ± SEM. Number of animals in parentheses. Comparison of the same parameter among groups: ANOVA (Hematocrit, p = 0.1807; Osmolality, p = 0.3577; Creatinine, p<0.006; Uric acid, p<0.0001; Urea, p<0.003; Protein, p<0.0001). Post hoc Student-Newman-Keuls (different letters over the bars indicate statistical differences: creatinine and urea, p<0.05; uric acid and protein, p<0.01).

Figure 1 shows that hematocrit and plasma osmolality did not differ among all examined groups. Allopurinol or *vCdt* alone, or the association of *vCdt* with probenecid caused hypercreatinemia, in comparison with control (ip+po), but envenomed animals treated with allopurinol had a mitigation of this hypercreatinemia. The rise of uricemia induced by *vCdt* was normalized by allopurinol and probenecid. Compared with control, allopurinol alone caused hypouremia. Allopurinol associated with *vCdt* caused hyperproteinemia.

**Figure 2:**
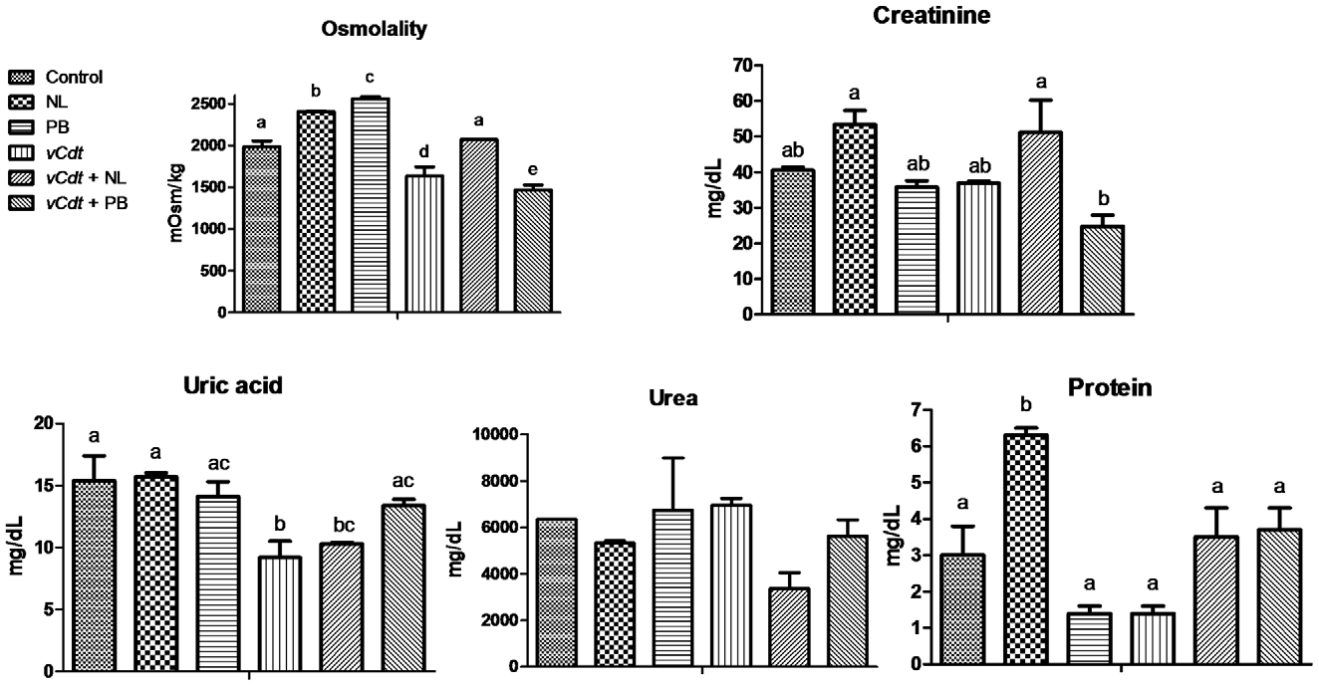
Effects of treatments on renal function parameters in the urine. Mice treated with vehicle (control), allopurinol (NL), probenecid (PB) and *Crotalus durissus terrificus* venom (*vCdt*) followed by NL (*vCdt*+NL) or PB (*vCdt*+PB) after 2 h. Values are means ± SEM of pooled animals, 16 (control), 9 (NL), 10 (PB), 12 (*vCdt*), 22 (*vCdt*+NL) and 12 (*vCdt*+PB) in triplicates. Comparison of the same parameter among groups: ANOVA (Osmolality, p<0.0001; Creatinine, p<0.0009; Uric acid, p<0.005; Urea, p = 0.06; Protein, p<0.001). Post hoc Student-Newman-Keuls (different letters over the bars indicate statistical differences: osmolality and creatinine, p<0.01; uric acid and protein, p<0.05).

Figure 2 shows that urinary urea was not affected by the treatments under study. Relative to control, urinary osmolality and urinary content of uric acid were diminished by *vCdt*. Allopurinol and probenecid alone caused a rise in urinary osmolality, but only the treatment of envenomed animals with allopurinol restored this parameter to a level of control group. Urinary content of uric acid in envenomed animals was normalized only by treatment with probenecid. The association of probenecid and *vCdt* caused hypocreatinuria. Allopurinol alone caused hyperproteinuria.

### Oxidative stress

**Figure 3:**
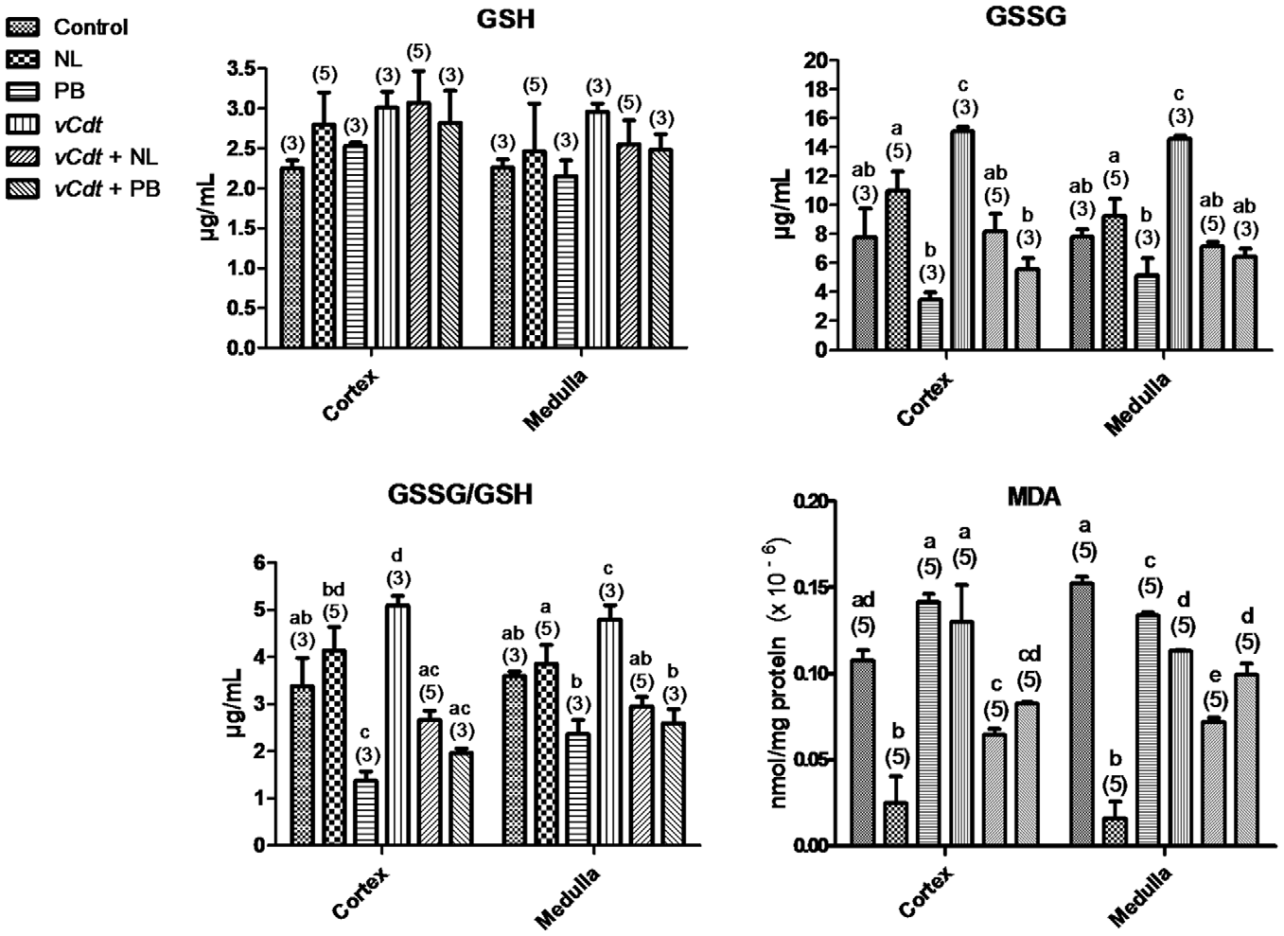
Effects of treatments on renal oxidative stress. Reduced glutathione (GSH), oxidized glutathione (GSSG) and malondialdehyde (MDA) were measured in renal cortex and medulla from mice treated with vehicle (control), allopurinol (NL), probenecid (PB) and *Crotalus durissus terrificus* venom (*vCdt*) followed by NL (*vCdt*+NL) or PB (*vCdt*+PB) after 2 h. Values are means ± SEM. Number of animals in parentheses. Comparison of the same parameter among groups: ANOVA (cortex: GSH, p = 0.6489; GSSG, p<0.0004; GSSG/GSH, p<0.0001; MDA, p<0.0001; medulla: GSH, p = 0.8440; GSSG, p<0.0001; GSSG/GSH, p<0.0009; MDA, p<0.0001). Post hoc Student-Newman-Keuls (different letters over the bars indicate statistical differences: p<0.05).

Figure 3 shows that the pattern of changes on GSH, GSSG and GSSG/GSH ratio caused by all the treatments under study was similar between the renal cortex and medulla, in comparison with control (ip+po). GSH was not altered by any treatment under study. GSSG and GSSG/GSH ratio were increased by *vCdt*. Probenecid diminished GSSG/GSH ratio in non-envenomed and both allopurinol and probenecid normalized GSSG and GSSG/GSH ratio in envenomed mice. Probenecid and *vCdt* did not affect MDA in the renal cortex but slightly diminished MDA in the renal medulla. Allopurinol decreased MDA in the renal cortex and medulla in envenomed and markedly in normal healthy mice.

### Histology

**Figure 4:**
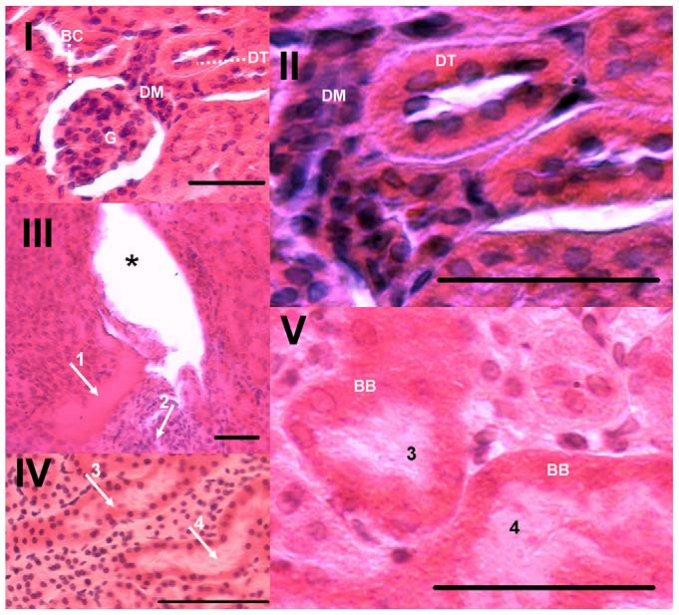
Histopathological features of renal samples. Slides of hematoxilin-eosin stained sagittal sections from representative kidneys of mice treated with: **I** and **II**: vehicle (control); see normal appearance of Bowman's capsule (**BC**), glomerulus (**G**), macula densa (**DM**) and distal tubule (**DT**); **III**, **IV** and **V**: *C. d. terrificus* venom; the most frequently changes detected were edema (*), fibrosis (**1**) with cell influx (**2**), and tubular necrosis with tubules markedly dilated and cellular debris in the lumen (**3**, **4**) and in the brush border (**BB**). Treatments of envenomed with allopurinol or probenecid acted indistinguishably to ameliorate the frequency and the intensity of detectable histological changes comparatively with untreated envenomed mice. Bars = 100 µm.

Histopathological changes such as edema, fibrosis and tubular necrosis were observed in envenomed mice, corroborating previous findings about direct nephrotoxic effects of this venom [5], [32], [36]. These changes were predominant in the cortex. The kidneys of animals treated with allopurinol or probenecid have much less intense and less numerous alterations, with an appearance that resembles that of the control animals. Some of these histological aspects were illustrated in Figure 4.

## Discussion

ARF induced by *vCdt* through direct renal action in mice promotes histological changes, hyperuricemia, hypercreatinemia, increased renal GSSG/GSH ratio with unaltered lipidic peroxidation, hypo-osmolality, decreased excretion of uric acid in urine and death after a time course higher than 14 h. Epidemiological data compiling reports of snakebites in Brazil during 100 years show that the average time elapsed between snakebites in humans and the medical attendance is generally less than 6 hours [37]. In the case of envenomation by rattlesnake, urinary changes do not usually occur before the 12th hour [2] and the available data on the distribution of the number of attendances, according to the average time elapsed between the accident and the attendance is about 67% (<3 h), 14% (3–6 h), 11% (>6 h), 8% (unknown) [38]. In the present study allopurinol and probenecid administered 2 h after *vCdt* fully restore uricemia and renal GSSG/GSH ratio and ameliorate histopathological changes caused by this venom. Additionally, probenecid restores uricosuria, while allopurinol restores the normal levels of urinary osmolality in envenomed mice. Above all, allopurinol significantly decreases the lethality of *vCdt*. This is the first study correlating agents capable of reducing renal uric acid with the ARF induced by animal venoms.

A typical property of allopurinol is to decrease the concentration of uric acid and urates relatively insoluble in tissues, plasma and urine. Allopurinol blocks the formation of uric acid, reducing its synthesis by competitive inhibition of xanthine oxidase [39]. Allopurinol is rapidly and extensively metabolised to oxypurinol, and its hypouricemic efficacy is due very largely to this metabolite [39]. Thus, its beneficial effect on envenomation by *vCdt* should be associated to the cell lysis caused by this venom, which contributes to the formation of uric acid and consequent deposit of urate. On the other hand, probenecid is an inducer of uric acid excretion in urine, without influence on its formation [40], acting as an inhibitor of an organic anion transport exchanger that blocks the entry of uric acid into the cells [41]–[42]. Allopurinol is approved by the US Food and Drug Administration for a dose up to 800 mg/day and is available as a low-cost generic drug [43]. Allopurinol at the same dose used in the present study and administered orally for 7 days possess potent hypouricemic effect in mice [44]. Probenecid at the same dose and route used in the present study accelerates the uric acid excretion in mice [45]. A single dose of allopurinol by the same route and thirty times lower than that used here is effective as uricostatic [46], while a fiftieth part of a single dose of probenecid by the same route used in the present study promotes a consistent uricosuric response in human subjects [47]. The same dose of allopurinol used here, but administered by intra-arterial infusion, totally abolishes the vascular oxidative stress [40], while about a half of this dose administered by the same route improves peripheral vasodilator capacity and blood flow in humans with chronic heart failure [48]. In the present study, despite having played its excretory activity in envenomed mice, probenecid did not provide the same beneficial effect of allopurinol against the lethality (it tended to reduce mortality, but without statistical significance). Furthermore, a rise of the antioxidant uric acid in the plasma paradoxically induced increased GSSG/GSH ratio and unaltered level of MDA in kidneys of mice envenomed by *vCdt*; and allopurinol and probenecid were both efficient in restoring this GSSG/GSH ratio to normal value. Other studies have reported that simvastatin [6] and lipoic acid [7] also ameliorated the GSSG/GSH ratio in the renal tissue, but these drugs did not affect the lethality caused by *vCdt* in mice. Thus, only the reduction of this index of oxidative stress was not responsible for the reduction of the lethality of *vCdt*. Furthermore, the antioxidant effect of simvastatin [6], lipoic acid [7], allopurinol and probenecid, as well as the lethality of *vCdt* could not be related simply to a uric acid-associated crystal-dependent mechanism. Therefore, what are the additional beneficial effects of allopurinol in this envenomation?

**Figure 5:**
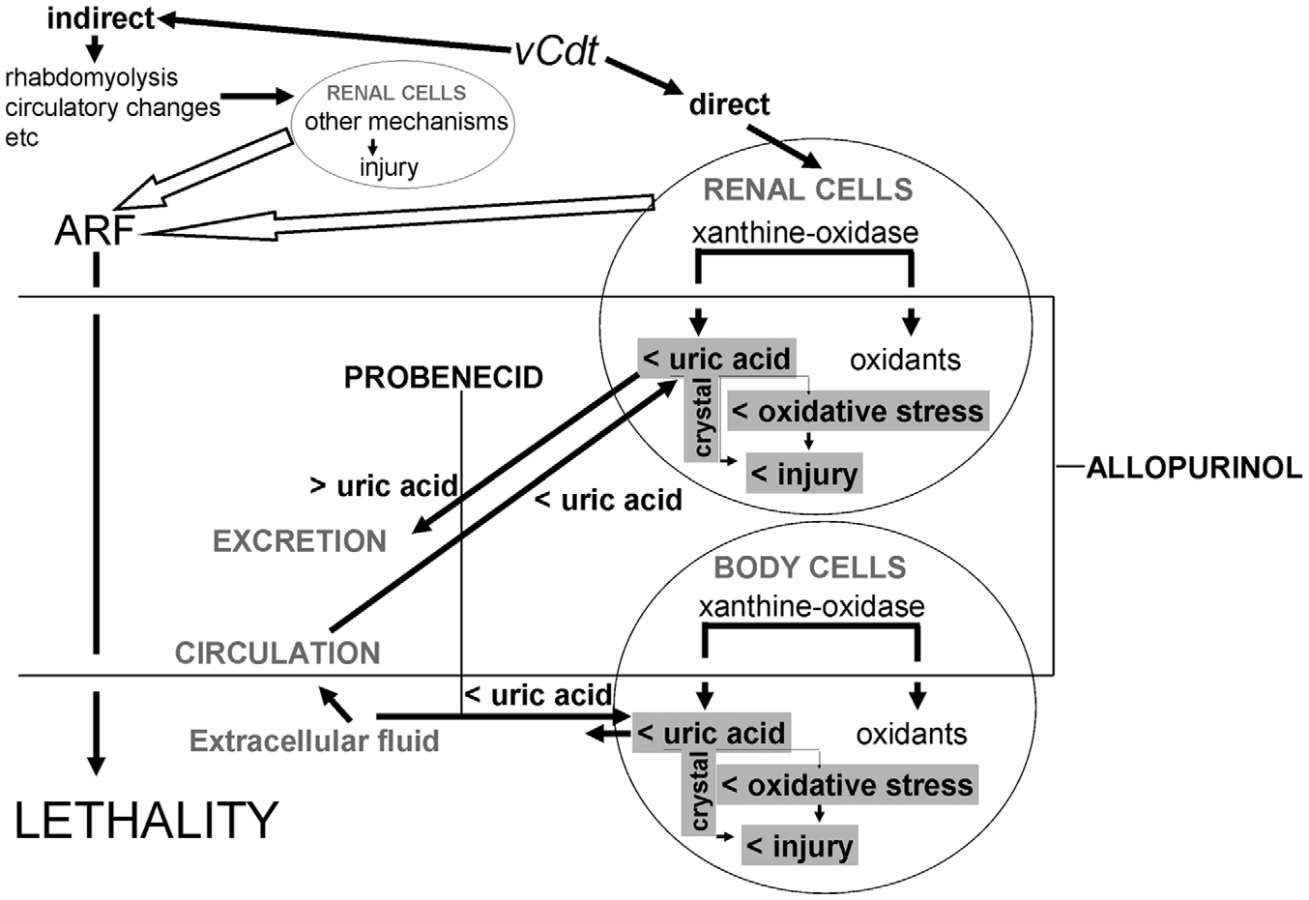
Schematic diagram depicting mechanisms and hypothetical actions of allopurinol and probenecid in *vCdt* nephrotoxicity. ARF induced by *vCdt* occurs through indirect and direct actions leading to lethality. Direct actions of *vCdt* generate hyperuricemia and renal oxidative stress. Uric acid has direct intracellular pro-oxidative effects. Allopurinol and probenecid restore uricemia and renal oxidative stress caused by *vCdt*. These beneficial effects must be, in part, exerted through a reduction of intracellular deposit of urate, as a consequence of the reduction of uric acid formation due to the inhibition of xanthine oxidase (allopurinol) or an inhibition of an organic anion transport exchanger that blocks the entry of uric acid into the cells (probenecid). Allopurinol, but not probenecid, protects against the lethality caused by *vCdt*. This differential protective effect of allopurinol is not related to the blocking of xanthine oxidase-associated oxidants, but it is likely related to the blocking of oxidant effects of increased production of uric acid in the intracellular environment more than the entry of uric acid into the cells.

Allopurinol could be protective due to the blocking of xanthine oxidase-associated oxidants [49] as opposed to the simple uric acid-lowering effect of probenecid, since the pathway of xanthine oxidase, which leads to the synthesis of uric acid, can form reactive oxygen species such as MDA [35], which can attack a wide variety of cellular components. In fact, the present study shows that besides reducing the uric acid, allopurinol was active on MDA-mediated lipid peroxidation in the kidney. However, we did not observe any significant changes in lipid peroxidation expressed by MDA level in envenomed mice as compared with healthy controls. Allopurinol at a dose twice lower has reported to be more effective than probenecid in improving endothelial function in patients with congestive heart failure, despite equivalent lowering of uric acid [40]; and in the present study the ratio between the doses of allopurinol and probenecid was 1∶12. It is known that uric acid itself may cause endothelial dysfunction, which requires intracellular uptake of uric acid (as noted by the ability of probenecid to block the effects of uric acid on vascular cells) [21]. In this regard, allopurinol may be more effective at lowering intracellular uric acid levels when cellular production is high such as observed in heart failure [40]. These findings suggest that increased cellular production of uric acid is an important cause of the hyperuricemia induced by *vCdt*. Furthermore, uric acid, while being an antioxidant in the extracellular environment, has direct pro-oxidative effects once it gains entry into cells [30]. When uric acid reacts with oxidants such as peroxynitrite, it generates both radicals and alkylating species as it degrades peroxynitrite [50]. In the extracellular environment, these substances may dissipate into the circulation, but these substances are highly likely to be reactive with local constituents in the intracellular environment. In addition, uric acid may also be more likely to function as an antioxidant in a hydrophilic environment (such as present in the extracellular environment) as opposed to the primarily hydrophobic intracellular environment [51]. On the other hand, it is well documented that probenecid reduces the uric acid excretion rate at a low dose and accelerates it at a high dose, showing the so-called paradoxical effect [45]. The differential determinants of the low efficiency of probenecid compared to allopurinol against the lethality of *vCdt* could be associated with this paradoxical effect and/or with effects that occur only with the administration in envenomed animals. In fact, this last kind of effect of allopurinol is only the hyperproteinemia, while probenecid promotes hypercreatinemia, hypocreatinuria and aggravation of the urinary hypo-osmolality in envenomed mice. Based on this framework of evidences, the most likely mechanisms of differential action of allopurinol and probenecid on the lethality induced by *vCdt* are sketched in Figure 5.

In conclusion, the evidences presented here support the hypothesis proposed by Yamasaki et al. [6] that the hyperuricemia is involved in the early stages of ARF induced by direct nephrotoxic action of *vCdt*. Data shows that this envenoming constitutes an attractive animal model suitable for studying the hyperuricemia and that the therapeutic intervention with allopurinol at an early stage can prevent or recover its renal effects and especially prevent its lethality in mice. While one must be cautious in extrapolating animal models to human disease, this study provides a consistent evidence linking uric acid with the ARF induced by *vCdt* and should stimulate clinical trials to address whether allopurinol may contribute to anti-snake venom serotherapy.
